# Greenhouse Studies of Thiamethoxam Effects on Pea Leaf Weevil, *Sitona lineatus*

**DOI:** 10.1673/031.012.15101

**Published:** 2012-12-28

**Authors:** Héctor Cárcamo, Carolyn Herle, Vincent Hervet

**Affiliations:** ^1^ Agriculture and Agri-Food Canada, Lethbridge Research Centre, 5403-1st Avenue South, Lethbridge, Alberta, Canada T1J 4B1; ^2^ Institut Polytechnique LaSalle Beauvais, 17 rue Pierre Waguet 60000 Beauvais Current Address: Department of Biological Sciences, University of Lethbridge, Lethbridge, Alberta, Canada T1K 3M4

**Keywords:** Broad nosed weevils, insecticide, IPM, neonicotinoids, *Pisum sativum*, seed

## Abstract

The pea leaf weevil, *Sitona lineatus* L. (Coleoptera: Curculionidae), has recently emerged as an important pest of field peas in the Canadian prairies. Systemic seed-coated insecticides may provide a tool for the integrated pest management of this pest. Therefore, several controlled assays were performed in order to determine effects of a recently registered neonicotinoid, (thiamethoxam) on *S. lineatus* damage to foliage, weevil mortality, fertility, egg viability, larval mortality, and root nodule damage. Foliage damage was reduced by thiamethoxam relative to untreated controls during the seedling stage (2^nd^–5^th^ nodes), but weevil adult mortality was only 15–30%. Fertility was reduced substantially through an extra seven-day delay in the preoviposition period and reduced egg-laying rate during the first 20 days of the study (92% lower than controls). Overall egg viability was lower in females fed foliage grown from thiamethoxamtreated seeds. Larval survivorship and nodule damage were also lower, but only when eggs were added to treated plants at the 2^nd^ node stage. When eggs were added late, at the 5th node stage, thiamethoxam had no effect on larval survivorship or nodule damage. The results of this study led to the conclusion that seed treatments such as thiamethoxam have potential to be used as tools that will aid in the integrated pest management of *S. lineatus*, especially in combination with other methods such as biocontrol and trap crops.

## Introduction

The pea leaf weevil, *Sitona lineatus* L. (Coleoptera: Curculionidae), is a common pest of peas (*Pisum sativum* L (Fabales: Fabaceae) and faba beans (*Vicia faba* L.) in Europe, North Africa, and North America. It has recently expanded its range to the Canadian Prairies, a significant pea production area (Vankosky et al. 2009). The life cycle of *Sitona* weevils was described in detail by Jackson (1920). Adults of *S. lineatus* overwinter in perennial legume crops, field margins, or shelters, then migrate to pea fields in the spring and produce characteristic Ushaped notches on foliage. Within a few days, females scatter their eggs on the soil surface around pea seedlings and continue ovipositing throughout the summer. Larvae feed on *Rhizobium* root nodules, which can potentially reduce yield if soil is nitrogen deficient (Cárcamo and Vankosky 2011). The immature stages last 6–8 weeks, and adults of the new generation emerge in mid-late summer to continue feeding on any green foliage of plants of the Fabaceae family.

Managing *S. lineatus* has relied on foliar or seed-coated insecticides with variable results. In general, foliar insecticides do not seem to provide sufficient control to prevent larval damage to nodules, despite reductions in adult foliage consumption (Seidenglanz et al. 2010; Cárcamo and Vankosky 2011). A number of seed-coated or soil-incorporated systemic insecticides have been used with some degree of success. For example, Bardner et al. (1983) and Lee and Upton (1992) suggested that phorate and bendiocarb, respectively, may improve yield of peas when *S. lineatus* reach pest levels. A summary of the 13 chemicals used for this pest was provided by Vankosky et al. (2009), and again suggested variable success in controlling this pest. Thiamethoxam is a neonicotinoid seed-coated insecticide recently registered for *S. lineatus* in field peas in Canada (Government of Alberta 2011), initially as an emergency registration to provide growers with an option to manage this pest. Thiamethoxam is efficient in controlling leaf hoppers in potatoes (Nault et al. 2004) and provided sufficient crop protection against wireworms in cereals (Vernon et al. 2009). However, the mechanism of action with respect to its efficacy for *S. lineatus* other than reduction of foliage damage remained unknown. For example, insecticide expression in the roots and nodules and effects on larvae are poorly known. *Rhizobium* nodules vary in size and physiological status as they age (Groten et al. 2005). Higher number of larger, tumescent (branched) nodules in plants with insecticide seed treatment may indicate higher nodule survivorship in those plants.

Furthermore, studies of this and similar neonicotineoid insecticides have suggested selective mortality or sub-lethal effects on insect fitness. For example, a related compound, imidacloprid, increased the fertility of *Tetranychus urticae* Koch and may explain increases in populations of this mite when this insecticide was sprayed against psyllids on pears (Beer and Himmel 2002). Therefore, in the context of assessing this registered seed treatment as a tool to improve the integrated pest management of *S. lineatus*, several greenhouse and laboratory studies were conducted in order to determine the effects of thiamethoxam on adult damage to pea plants, weevil mortality and fecundity, egg viability, larval mortality, and damage to root nodules.

## Materials and Methods

### Insect stock

Weevils for Tests 1 and 2 were field collected in the spring and summer of 2008, respectively, from plots of field peas in the vicinity of Lethbridge, Alberta, Canada. Insects were picked off the ground around pea plants and placed in 0.5 L plastic containers with pea foliage and kept at room temperature until required. Tests 3 and 4 were conducted in the spring of 2009 using weevils that were collected in high numbers on a combine table while a farmer combined his pea field near Vulcan, Alberta, in September 2008. These weevils were fed alfalfa for two weeks prior to being overwintered at 5° C until 3 February 2009, when they were brought to room temperature and fed sprouted alfalfa.

For Test 5, conducted in 2010, weevil stock was obtained by overwintering adults collected on 24 August 2009 from field tests in cages with pea plants grown in 2009. These weevils were fed alfalfa for two weeks prior to overwintering at 5° C. They were transferred from 5° C to 20° C on 7 January 2010 and fed alfalfa for one week.

### Foliage damage and adult mortality tests

A series of assays, replicated at least 10 times, using *S. lineatus* adults were performed in a greenhouse in order to assess the effects of thiamethoxam on foliage damage, adult mortality, reproductive potential, and egg viability. All seed used in all tests (1–5) was treated with Apron Max® (6.5 g/100 kg seed), a fungicide package for various canola seedling diseases (Syngenta Canada Inc., http://www.syngenta.com/). During the day of planting, the peat inoculant N Prove containing *Rhizobium leguminosarum* (7.4 × 10^8^ viable cells per gram from Novozyme Biologicals, http://www.novozymes.com/) was applied to pea seed of all treatments (except treatment 1 of Test 1) at a rate of 0.17 g per 100 g of pea seed. Although cultivars varied among some of the trials, no plant genetic effects were expected or observed, as host plant resistance is rare for this insect pest (see review by Vankosky et al. 2009).

Test 1 was designed to determine thiamethoxam insecticide dose effects on pea foliage damage. Two males and two females were added to a pot containing two pea plants (or one pair of weevils if only one plant survived) confined with a sleeve mesh cage constructed with Dacron Chiffon (Bioquip, http://www.bioquip.com/; Product 7250C). The six treatments tested are listed in Table 1. In Test 2, the effect of plant density and insecticide treatment on foliage damage was tested. A similar meshed cage design was used in this test, but the density of plants per cage (2, 4, and 8) was manipulated using a control of untreated seed and a thiamethoxam (30 g a.i./100 kg seed, Syngenta Canada Inc.). Two cohorts of weevils were used. The first was old reproductive weevils collected in early August and added to plants at the second node stage on 5 August 2008. Weevils were removed after 48 hours and transferred to Petri dishes, where they were fed untreated foliage to determine their mortality. The second batch of weevils was added around the 4–5^th^ node stage using young weevils of the new generation that were collected from pea plots and added to the cages on 11 August 2008. Again, they were allowed to feed for 48 hours and later fed untreated foliage in Petri dishes. Foliage damage caused by each cohort was recorded, and mortality of adults in Petri dishes was noted.

**Table 1.**
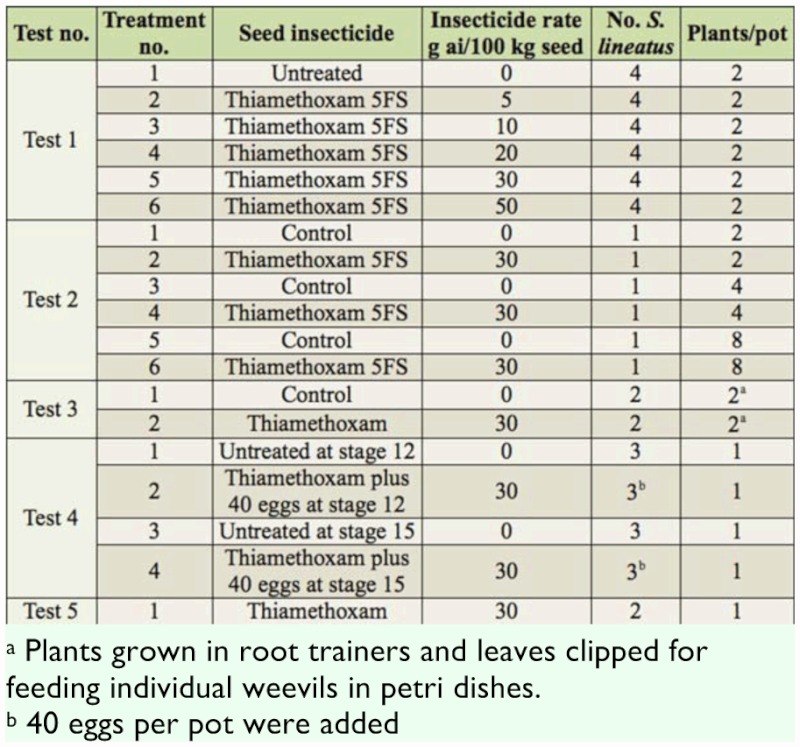
Summary of the various greenhouse experiments conducted during 2008–2010 to assess effects of thiamethoxam on *S. lineatus* in field peas. Ten replicates were completed except for Tests 3 (30) and Test 5 (40). Test number, period, and variety: Test 1: 04/06/08 – 22/08/08 Cutlass: insecticide dose effects on pea foliage damage; Test 2: 25/07/08 – 13/08/08 Cutlass: effect of plant density and insecticide on foliage damage; Test 3: 14/03/09 – 24/04/09 Capri : insecticide effects on weevil fertility; Test 4: 12/03/09 – 13/05/09 Capri: insecticide and crop stage interaction; Test 5: 04/02/10 – 30/03/10 Thunderbird: mortality re- assessment.

In Test 3 (insecticide effects on weevil fertility), the number of eggs laid by weevils isolated in male/female pairs in Petri dishes and fed either foliage grown from treated or untreated seed were measured. Pea plants were grown in root trainer pots (3.5 cm × 11 cm) and four plants were allocated to provide foliage for each weevil pair for the 41-day study. The two youngest fully expanded leaves from each node were fed to the weevil pair. Dead weevils were replaced with one of the same sex. During each feeding, mortality, number of eggs, notches on stipules, and frass spots were counted. Foliage was visually rated for damage levels using a scale ranging from 0 to 3, with 0 = no feeding, 1 = few notches, 2 = several but distinct notches, and 3 = heavy, numerous and coalesced notches. At growth stages 12 and 14, the foliage was scanned using a flat bed scanner (Epson Perfection 4990) to determine quantitatively the amount consumed by weevils using image analysis (Image Pro Plus v4.1). A subsample of 225 and 201 eggs from the control and thiamethoxam treatments, respectively, were kept at 20° C and 18:6 L:D in snap-lock Petri dishes with moistened blotter paper to quantify hatchability three times per week. Eggs were kept in groups of 2–59 eggs per dish, but only those with at least 10 eggs were retained for statistical analysis.

In Test 3, weevil mortality was assessed by examining weevils found on their back for leg movement over a few minutes and declaring them dead if they stayed still on their backs with no leg movement. However, it was subsequently learned that in other beetle taxa (Elateridae; Vernon et al. 2009), such assessment is inadequate as insects may recover after some time. Therefore, to confirm mortality of weevils, Test 5 was performed (Table 1, “mortality re-assessment”). Plants were grown from thiamethoxam treated seed in 7.6 cm × 7.6 cm × 20 cm mini Tree pots (Stuewe and Sons, Tangent, http://www.stuewe.com/; Product MT38) individually and enclosed with clear Plexiglas tube. At the 2nd node stage, a male and female pair were added and observed daily during weekdays for two weeks. Individuals that appeared dead (i.e., on their back and motionless) were kept under observation for possible recovery for two weeks and carefully examined every 24 hours (except during weekends) under a microscope at 100× magnification for mandibular twitching as suggested by Robert Vernon, Agriculture and Agri-Food Canada (personal communication with H. Cárcamo).

### Larval mortality and nodule damage

Eggs from weevils fed untreated plants from the oviposition test described above were stored at 20° C (18:6 L:D) in snap-lock Petri dishes with moistened blotter paper and used to determine larval mortality and nodule damage in Test 4 (Table 1, “insecticide and crop stage interaction”). Groups of 40 eggs were added to mini Tree Pots containing one pea plant allocated to the following four treatments (replicated 10 times): 1) control plants at 2^nd^ node; 2) control at 5^th^ node stage; 3) thiamethoxam treated plants at 2^nd^ node; 4) thiamethoxam treated plants at 5^th^ node. Plants were harvested at the early pod stage around 31–35 days after stocking them with eggs. Total nodules were recorded according to their form (tumescent or single), number of protuberances in each node, damaged or not, and expression of laeghemoglobin (pink or not). The number of *S. lineatus* and their stage was noted (larva, pupa, adult).

### Statistical analysis

All foliage damage data were summarized on a per plant basis and subjected to Analysis of Variance using the Proc Mixed of SAS (version 9.2). Test 1 was analyzed using Repeated Measures to determine differences in damage at the two crop stages by the same weevils and possible interactions with the main effect (chemical treatment). For this test, Tukey's adjustment was used to separate means (*p* < 0.05) to account for the large number of treatments. In other tests with few treatments, FPLSD (*p* < 0.05) was used after ANOVA with SAS proc mixed indicated a significant model effect. Where necessary, data were transformed using log (× +1), or, for proportional data, the arc-sin transformation was used, but data are presented as raw means with one standard error. For weevil mortality data with very low counts and many zeros, analysis was performed on ranked data and means separated as above. For the egg viability study, only dishes with at least 10 eggs were kept for analysis of proportion of egg hatch after it was transformed using the Arcsine option in Systat v. 12.

**Table 2.**
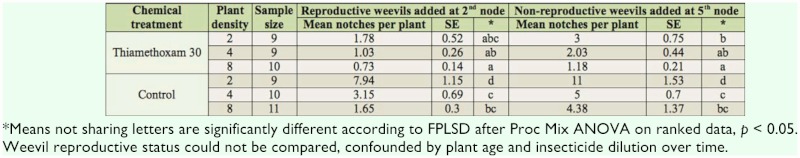
Foliage damage to peas by *S. lineatus* female confined over 48 hours with 2, 4, or 8 plants grown from thiamethoxamtreated seed or untreated.

## Results

### Foliage damage and adult mortality

**Figure 1.**
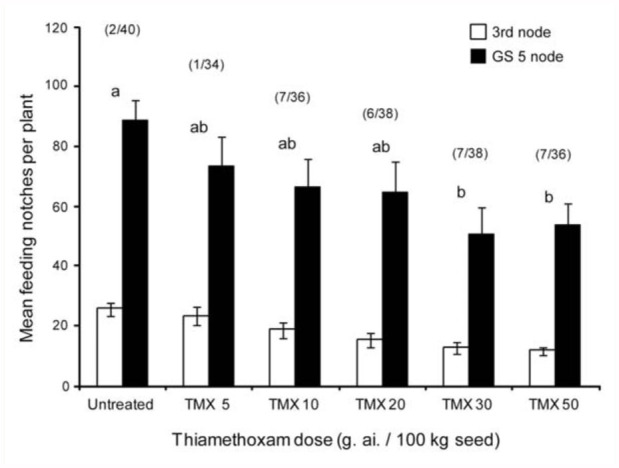
Effect of thiamethoxam dose (g a.i./kg seed) on foliage damage to peas (bars represent I standard error of the mean) and total *Sitona lineatus* mortality (weevils dead/total weevils per treatment). High quality figures are available online.

In Test 1, there was a significant insecticide treatment effect on the number of notches per plant and significantly more (3–4 times) feeding on plants at the 5^th^ node stage compared to the 3^rd^ node stage, but there was no interaction between treatment and crop stage (Figure 1). Only plants grown from seeds coated with at least 30 or 50 g a.i. of thiamethoxam had significantly fewer notches, at either crop stage, than those grown from untreated seed. The number of dead weevils (out of up to four individuals added per pot) was very low (Figure 1) and ranged from 0.1 (thiamethoxam -5 g rate) to 0.7 in the thiamethoxam (-50 g rate). Analysis of ranked data suggested that the highest rate of thiamethoxam (50 g) had significantly higher mortality than the control without insecticide and the lowest rate of thiamethoxam (5 g).

In Test 2, plant density and chemical treatment influenced the number of notches per plant but the two factors did not interact (Table 2). The number of notches per plant was lower in pots with 4 or 8 plants than in those with 2 plants, regardless of insecticide treatment. In every case, for each plant density, the plant damage was higher in the untreated seedlings compared to those in seedlings grown from seed coated with thiamethoxam. Mortality of adults attributed to insecticide seed coating was very low for the two weevil cohorts. For the old reproductive weevils added to the plants at the 2^nd^ node stage, the total mortality out of 10 weevils, three days after stocking, ranged from 1–2 in the controls and 3–4 in the pots with plants grown from seed coated with thiamethoxam. For the new generation nonreproductive weevils added at the 4–5^th^ node stage, mortality in the thiamethoxam treatment ranged from 0 (two-plant treatment) to 2 weevils and 1–2 in the controls. Given these low and variable numbers, no statistical analysis was performed for weevil mortality in this test nor could the differences between weevil cohorts be tested since age was confounded by crop stage and the lower insecticide potency expected in older plants at the 5^th^ node stage.

**Figure 2.**
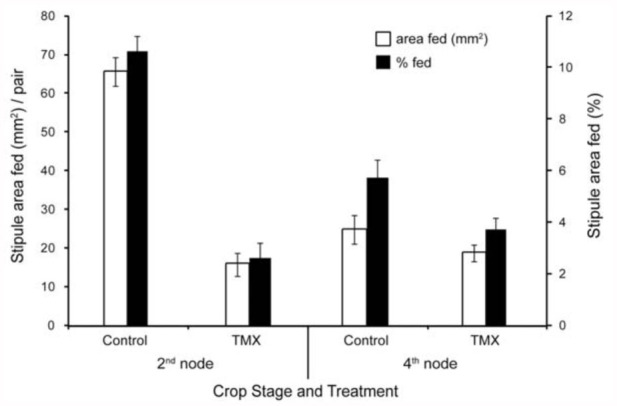
Effect of thiamethoxam on pea stipule consumption (area and percentage) by *Sitona lineatus* measured with image analysis at the 2^nd^ and 4^th^ node stages. High quality figures are available online.

Results from Test 3 agreed with the first two tests with respect to foliage damage by adult weevils. Plants from treated seed suffered significantly less feeding damage by *S. lineatus* than control plants. All measures of feeding, including number of notches per stipule, visual ratings of damage (data not shown), area and percentage of stipule consumed (measured by image analysis), and number of fecal spots on filter paper, were significantly higher for the control treatment than the thiamethoxam treatment for about 14 days of feeding (Table 3) and 2–3 times higher during the first 10 days. The subsample subjected to image analysis further demonstrated quantitatively the effect of thiamethoxam on stipule consumption (Figure 2). Differences were larger and statistically significant at the 2^nd^ node stage, when weevils consumed about 65 mm^2^ of untreated foliage, which equaled almost 11% of the area, in contrast to 16 mm^2^ of foliage from thiamethoxam treated seed which represented only about 3% of the area. At the 4^th^ node stage, the amount fed was 19–25 mm^2^ in the thiamethoxam and untreated foliage and did not differ statistically and represented only 36% of the stipule area.

**Table 3.**
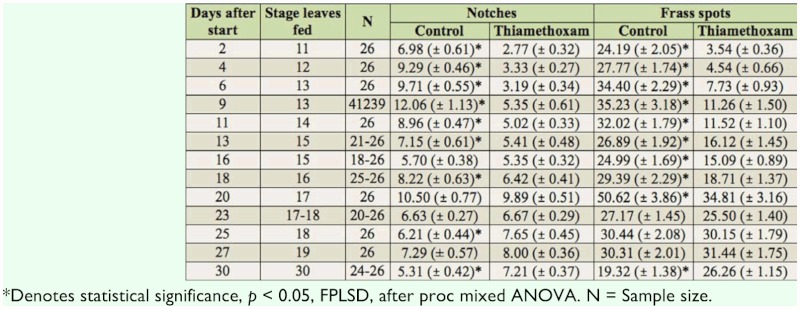
Effects of thiamethoxam (30 g a.i./100 kg seed) on number of notches, and number of frass spots in the laboratory study of *Sitona lineatus* fed field pea foliage from thiamethoxam or control plants in Petri dishes.

Weevil mortality in Test 3 was similar to the previous tests. Four females and 14 males were killed out of 30 pairs in the thiamethoxam treatment (∼25% mortality) and zero in the controls. Results from Test 5, where weevils were allowed to feed on thiamethoxam treated foliage on live plants from the 2^nd^–8^th^ node stage confirmed the low mortality of weevils (11 of 78). Within 24 hours of feeding, only 1 weevil died. After four days of feeding, 3 more adults died and 2 others were knocked down but recovered within 24 hours. The remaining 7 adults that died took 7–15 days to be killed.

### Oviposition

**Figure 3.**
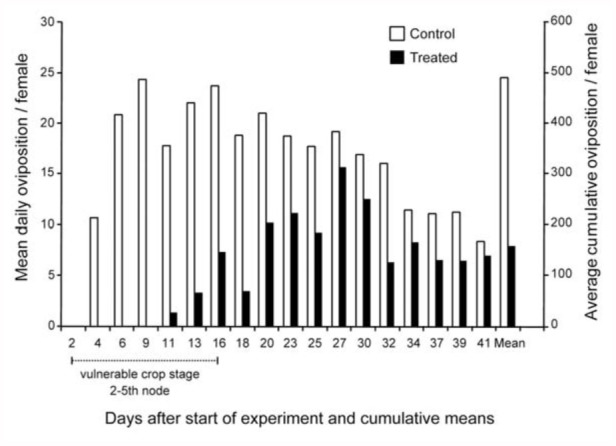
Effect of thiamethoxam on *Sitona lineatus* oviposition rates over a 41 -day study (entries are daily egg laying means for varying numbers of females) and the average total (cumulative) number of eggs laid per laying female. High quality figures are available online.

Thiamethoxam affected a number of *S. lineatus* oviposition parameters (Figure 3). Females fed pea foliage from thiamethoxamtreated seedlings had a pre-oviposition period of 11 days, whereas those fed control foliage had a pre-oviposition period of only four days. The average cumulative number of eggs laid per female during the 41-day period was significantly lower for weevils fed thiamethoxam foliage (158 eggs/female) than for the control weevils (490 eggs/female). The range of total oviposition was 9–997 and 15– 341 eggs per female for the control and thiamethoxam treatments, respectively. The average number of eggs laid per female during the growth stage period considered vulnerable to pest damage (2^nd^ to 5^th^ node) was 290.0 (se = 78.9) for the control group and 23.17 (se = 7.5) for the thiamethoxam group (F_1,8_ = 17.89, *p* = 0.0029, proc mixed in SAS). Oviposition rates for the control group started at 11 eggs per day per female and peaked at 24 on Day 9 of the experiment. Females kept laying between 15–20 eggs until Day 31, then dropped to 8 on Day 41. Females fed foliage from thiamethoxam treated plants started laying 1–10 eggs per day and peaked at 15 eggs on Day 26 and dropped to less than 10 by Day 31 and to 7 by Day 41. The number of eggs laid per day was numerically higher in the Control than the thiamethoxam treatment at all sampling periods, and significantly so during the first 20 days of the experiment and on Days 25 and 32 (*p* = 0.05; Figure 3).

**Table 4.**
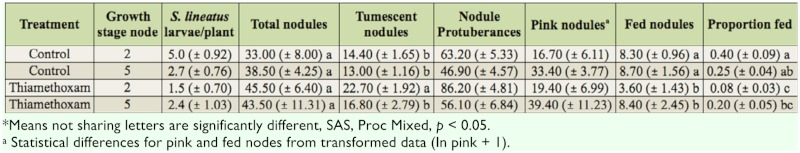
Effects of thiamethoxam and growth stage node of plant when eggs were added on root nodule parameters and *Sitona lineatus* larval survivorship after 40 eggs were added to each plant. Entries are means of 10 replicate pots and one standard error.

Overall proportion of viable eggs laid by weevils fed foliage from thiamethoxam and control treated plants was 73% and 97%, respectively. However, when dishes with at least 10 eggs were retained for statistical analysis, the average proportions were 84% and 96% for these treatments and did not differ significantly based on one-way ANOVA of arcsin transformed data (F_1,13_ = 1.17, *p* > 0.05).

### Larval mortality and nodule damage

In Test 4, insecticide treatment did not reduce the number of larvae recovered from pots if the eggs were added at the 5^th^ node stage, but there was a significant effect when they were added at the 2^nd^ node stage. Pots with plants grown from untreated seed with eggs added at the 2^nd^ node stage had about twice as many larvae than pots with plants treated with thiamethoxam from either crop stage (*p* < 0.05; Table 4). The total number of nodules per plant ranged from 33 to 45 and was not affected by treatment. However, the number of older tumescent nodules and average nodule protuberances were significantly higher in the thiamethoxam-treated plants than in the control plants, but only when eggs were added at the 2^nd^ node stage (Table 4). The number of fed nodules or the proportion of nodules damaged per plant was higher in the Control than in the thiamethoxam-treated plants only when eggs were added at the 2^nd^ node stage. The number of pink or active nodules was higher (2 times) in pots where eggs were added at the 5^th^ node than in those that received the eggs at the 2^nd^ node, but there was no chemical effect.

## Discussion

In the Canadian prairies of southern Alberta and southern Saskatchewan, *S. lineatus* has emerged as a new and potentially important pest of field peas (Vankosky et al. 2009), and several thousands of hectares have been sprayed since 2006 in years when adult weevil damage to seedlings has been high. In 2007, the seed-treatment thiamethoxam was registered on an emergency basis with the support of foliage-damage reduction data collected from other jurisdictions (T. Labun, Syngenta Canada Inc; personal communication with H. Cárcamo) and has since received permanent registration. However, prior to this study, it was not known what specific effects thiamethoxam had on egg, larval, and adult mortality.

The first test confirmed that the registered rate of thiamethoxam of 30 g a.i. per 100 kg of seed (∼ 82 g. of a.i./ha at the recommended seeding rate of 275 kg/ha as per Government of Saskatchewan Agriculture Knowledge, http://www.agriculture.gov.sk.ca/) had the lowest levels of feeding damage to stipules and was similar to the 50 g dose. These results are similar to those reported by Seidenglanz's et al. (2010) field study in the Czech Republic, who found no consistent differences in *S. lineatus* notching to unfolded pea stipules between a low dose (87.5 g of a.i./ha), equivalent to the registered rate in Canada, and a dose that was two-fold higher. The results of the present study also showed that using a lower rate would not result in a reduction of damage that differed from the untreated plants. The registered rate was effective at reducing foliage feeding by both reproductive and non-reproductive weevils.

The effect of thiamethoxam on feedingdamage reduction covers the duration of the crop stage considered vulnerable (2^nd^–5^th^ node). However, large differences in numbers of notches per plant in the range of 2–3-fold were only observed up to the 3^rd^–4^th^ node stage. This was confirmed by the more quantitative image analysis study, where small, non-significant, numerical daily reduction of 6 mm^2^ of treated and untreated foliage consumed by a pair of weevils at the 5^th^ node stage was found, in contrast to the 2^nd^ node stage. In a field study in southern Alberta, Canada, Vankosky et al. (2011) observed consistent reductions of foliage feeding by thiamethoxam up to the 5^th^ node stage and, in one of the three years, up to the 8^th^ node.

Mortality of adult weevils was consistently low in all the trials. All of the tests demonstrated that only 15–30% of adults that fed on pea foliage from thiamethoxam treated seeds (30 g/100 kg) actually died. Also, in contrast to reports on Elateridae where most are only intoxicated (Van Herk et al. 2008), this phenomenon seems to occur only in 15% of the knocked out weevils, and the remaining die. Mortality also occurred over a long period; 50% or more of the weevils took over a week to die. No previous studies have reported mortality of *Sitona* weevils to thiamethoxam, but a study on the carrot root weevil, *Listronotus oregonensis*, found lower yields of carrots with this seed-treatment compared to fipronil (Bonham et al. 2009), suggesting inconsistent control by the former compound. In a study of corn rootworm, *Diabrotica virgifera virgifera* Le Conte, Cox et al. (2007) found acceptable levels of yield protection with seed-treatments of thiamethoxam, similar to those reported in cereals with wireworms (Vernon et al. 2009). However, it is not known if rootworm s are actually being killed or simply intoxicated as is the case with wireworms (Van Herk et al. 2008). Similar inconsistent results were reported for *Aphis glycines* Matsumura in soybeans by Ohnesorg et al. (2009). In contrast, the potato leafhopper, *Empoasca fabae* Harris, is more susceptible to thiamethoxam, as shown by their significant reductions in density and plant-damage to snap bean grown from treated seed (Nault et al. 2004). It appears that neonicotinoid seedtreatments have selective, insect-killing properties, which may be a consideration in terms of conservation of natural enemies as pointed out by Ohnesorg et al. (2009), but sub-lethal effects on fitness need to be considered as well.

Over the 41 days in the oviposition study, a large reduction (67%) in reproductive potential of *S. lineatus* when fed pea foliage from plants treated with thiamethoxam was noted. This reduction was attributed to an increase of the pre-oviposition period by seven days, followed by a lower rate of egg laying throughout the study. In fact, over the first 20 days of the study, when plants were more vulnerable to pest damage, the reduction was 92% and may have had a positive effect on yield protection, depending on field populations and environmental conditions. The only published study assessing effects of thiamethoxam on pea yield in relation to *S. lineatus* feeding reported no effects on yield probably because nodule damage by larvae in that study ranged from 50–80% despite treatment with thiamethoxam (Vankosky et al. 2010). In an unpublished study assessing several insecticides in southern Alberta (Meers, unpublished data), thiamethoxam and other seed treatments appeared to improve yield relative to foliar compounds or controls, but field results seem inconsistent.

Sub-lethal effects of insecticides on arthropods, including fecundity and behavior, have been noted elsewhere (Croft and Brown 1975; Echegaray et al. 2008), including some studies on the relatively newer neonicotinoid class. For example, sublethal doses of Acetamiprid, but not thiamethoxam, affected honeybee behavior (El Hassani et al. 2007). Imidacloprid has been shown to increase the fecundity of *Tetranychus urticae* Koch under laboratory assays (James and Price 2002) and may explain the increase in populations of this pest when this insecticide is sprayed against psyllids on pears (Beer and Himmel 2002). In another study, thiamethoxam and other neonicotinoids at field-relevant doses reduced both survivorship of *T. urticae* and fecundity, though only very slightly relative to controls (Ado et al. 2004); sub-lethal doses had no effects. Effects of pesticides on life history characteristics of pests and beneficial arthropods are complex and should be researched in more systems.

*S. lineatus* larva suffered higher mortality than the adults in relation to thiamethoxam in the present study, but only ∼5 larvae survived per pot, which seemed low relative to the 40 eggs added at the start, but consistent with other investigations (Lee and Upton 1992). Crop stage was clearly an important determinant of larval survivorship and subsequent effects on nodule damage. A reduction in larval numbers and damage to nodules as well as an increase in the number of more complex (tumescent) nodules in relation to thiamethoxam treatment was only observed in the plants that received the eggs at the 2^nd^ node stage in contrast to those that received them at the 5^th^ node. This finding is important because it shows that growers should not need to take control action for early seeded crops when the weevils arrive past the 5^th^ node. This is the first study to present data on *S. lineatus* larval mortality in relation to thiamethoxam, which suggests activity of the chemical in the nodules; Seidenglanz et al. (2010) also reported an effect on larval mortality but neglected to present the data.

Effects of neonicotinoid insecticides on enhanced nodulation of peas or seedling vigor in general have been noted in other studies. Seidenglanz et al. (2010) reported an increase in total nodulation in the range of 43–363% from a number of compounds that included thiamethoxam, imidacloprid, and chothianidin. More modest nodulation increases (30–100%) in relation to thiamethoxam were reported by Vankosky et al. (2011). The mechanism for this effect is unknown, but some have speculated that thiamethoxam may stimulate greater formation of fine root hairs, which may harbor more *Rhizobium* bacteria (Vankosky et al. 2011). Higher root biomass has also been noted in crops such as canola in relation to neonicotinoid seed treatment (Tansey et al 2008). Increases in nodulation, however, may not translate to yield protection. In the study by Vankosky et al (2011), there was no effect of seed treatment on yield, which may be explained by incomplete control of the weevil or alternatively by lack of strong effects of the weevil on plant yield. In the study by Seidenglanz (2010), yields were not reported, but damaged nodules were not affected by treatments or were higher in the plots with thiamethoxam or another seed-treatment compared to the controls.

In conclusion, the present greenhouse and laboratory studies have elucidated the mechanism of the effect of thiamethoxam on *S. lineatus*; a small proportion of the adults (15–30%) are knocked down and the majority of these die (85%). The remaining weevils will lay substantially fewer eggs. Of the larvae that hatch and find nodules, only 30% are expected to survive in the thiamethoxam treated plants, provided the adults arrived and laid eggs during the early growth stages (2^nd^ node). The present study did not investigate effects on yield, but published and unpublished reports suggest inconsistent effects of thiamethoxam on yield protection. Seed treatments should be integrated with non-chemical strategies such as biological control or agronomic control to improve management of this pest.
